# A systematic approach to cancer: evolution beyond selection

**DOI:** 10.1186/s40169-016-0131-4

**Published:** 2017-01-03

**Authors:** William B. Miller, John S. Torday

**Affiliations:** 1 Independent Researcher, Paradise Valley, AZ 85253 USA; 2 Harbor-UCLA Medical Center, Torrance, CA 90502 USA

**Keywords:** Cancer, Self-reference, Information fields, Hologenome, Niche construction, Stigmergy, Aneuploidy, Tissue ecology, Cellular cognition, Phenotype

## Abstract

Cancer is typically scrutinized as a pathological process characterized by chromosomal aberrations and clonal expansion subject to stochastic Darwinian selection within adaptive cellular ecosystems. Cognition based evolution is suggested as an alternative approach to cancer development and progression in which neoplastic cells of differing karyotypes and cellular lineages are assessed as self-referential agencies with purposive participation within tissue microenvironments. As distinct self-aware entities, neoplastic cells occupy unique participant/observer status within tissue ecologies. In consequence, neoplastic proliferation by clonal lineages is enhanced by the advantaged utilization of ecological resources through flexible re-connection with progenitor evolutionary stages.

## Background

Since the 1970s, neoplasia has often been considered within an evolutionary framework [1, 2]. The current perspective considers cancer as selection biased differential fitness between heterogeneous populations of clonal lineages that arises as a function of genetic mutation and epigenetic alterations [3]. More recently, particular emphasis has been placed upon those features of cancer progression that focus on cancer as a participant in localized microenvironments that it partially creates [4, 5].

In nearly all previous discussions of the evolution of cancer, it is assumed that Darwinian fitness drives clonal selection through stochastic genetic variations that promote selective microenvironments [6, 7]. However, a different evolutionary narrative can be presented that productively recasts that prevailing model. Recent studies have highlighted the essential aspect of self-referential cognition in evolutionary development [8–10]. When that is honored, the exchange of information among self-aware constituencies within localized and distant tissue ecologies becomes the crucial element in an alternative evolutionary framework pertinent to cancer dynamics in which both are viewed as an uninterrupted cellular problem-solving process at every scope and scale in repeated reaction to environmental and epigenetic stresses [11–14]. When cancer is viewed within this frame, selection still pertains but its exact role and scope can be understood as secondary to a greater imperative of self-referential cells encountering stresses and reacting to them according to their limits. A productive reconsideration of the development of cancer and its proliferation enumerates from this vantage and energizes an exploration of the protean nature of neoplasia from within its root components and actual evolutionary mechanisms.

## Beyond Darwin

### Cognition based evolution (CBE)

In any productive consideration of cancer from an evolutionary perspective, certain elements are necessary aspects. All multicellular eukaryotes are holobionts [11, 15]. Each of these organisms are best understood as complex collaborative cellular partnerships that exist among a vast array of microbial life and the innate cells of any eukaryotic organism forming extensive mixed cellular ecologies [11]. Our understanding of that vital microbial fraction of any holobiont and its influence on it continues to expand. It is now apparent that any macroorganism should be evaluated as collaborative cellular entities in its response to stress and epiphenomena [16].

Cancer occurrence is widespread across multicellular eukaryotes. It is common in virtually all animals, though less so in plants and fungi. Only a few multicellular organisms are known to be highly cancer resistant, such as the naked mole rat [17]. Therefore, cancer can be considered as an essentially universal biological process occurring in multicellular eukaryotic organisms. Furthermore, cancer incidence occurs in organisms that remain intrinsically cellular in their respective responses to the environment across evolutionary development as holobionic life forms despite their macroscopic phenotypic appearances. Therefore, it is appropriate to assert that cancer that occurs in holobionic and intrinsically cellular life, as the exclusive macroscopic form on the planet, must represent a derivative cellular process whose evolutionary unfolding should center within similar cellular contexts.

An extensive body of evidence demonstrates that cognition is invested within all living things at every scope and scale [18–20]. It is now accepted that self-referential awareness of status is the conditional aspect of life and an innate property of all cells [10, 20]. This self-referential capacity is the means by which all cells assess homeostasis and attempt to maintain preferential states. However, prokaryotes and all individual cells have sets of faculties that extend beyond sustaining homeostasis as merely chemical interchanges and permit basal cognition that include aspects of storage of information as memory, learning, perception, and decision-making [19, 21, 22].

This level of self-referential awareness, in which cells have the direct ability to discriminate between states and direct actions towards one status compared to another, permits an extraordinary range of metabolic responses to stresses. In prokaryotes, this yields the interdependent, cooperative, and mutually competitive colonial form (biofilm). This ecology is sustained through the exchange of information utilizing sophisticated cell–cell communication [23, 24] and is similarly represented across eukaryotic mixed cellular ecologies. Survival and preferential status are advanced through swarming behaviors or cellular ecologies through the use of proxies, forms of memory and sociality [19, 25–28]. All of these sophisticated faculties are directed towards information processing and problem-solving [29–34] (Table 1).

**Table 1:** Summary of the cognitive faculties of cells

Individual perception/sensing [20, 23, 29]
Collective sensing/cooperation [25–28]
Complex communication [23, 24, 27, 28]
Autoduction/indirect sensing through proxies [19, 32]
Memory/information storage [20, 28]
Learning/behavioral adaptation [28–31]
Anticipation/prediction [22, 27]
Computation [34]
Directional motility [32]
Combinatorial decision-making/problem-solving [20, 28]
Trading of resources [26, 27, 33]
Sociality [19]

Such cellular cognition is surely limited but it does represent purposive use of information and communication, and as such, can be considered as a definitional aspect of life [10, 18]. This basal faculty is represented across the cellular sphere, be it microbial or the mixed cellular ecologies that comprise multicellular eukaryotic organisms. Therefore, it is appropriate to assume that cancer cells would be invested with the same property of self-referential awareness that is exhibited by all other cells. Further yet, cancer cells utilize this full range of general cellular mechanisms in their own unique manner to sustain their independent self-referential homeostatic status.

It is recognized that cancer is a distinct cell lineage with abnormal karyotypes as aneuploidy [35]. These karyotypes vary over a wide range and have been associated with chromosome mis-segregation that may yield differing phenotypes through chromosomal instability (CIN) [36].

However, aneuploidy is not always associated with chromosome mis-segregation or CIN, as both can occur in normal tissues across many species [37]. In fact, although aneuploidy is a feature in virtually all cancers, it has been also been shown to be an effective inhibitor of tumorigenesis under certain conditions [38]. Therefore, it can be inferred that although aneuploid cells are typical in cancer, they are context-specific as to site or tissue of origin within complex cellular ecological systems. When participating in tumorigenesis, such cells are participants in complex ecosystems in which clonal lineages of neoplastic cells compete and cooperate with each other and differing cells and other constituents within localized microenvironments [4]. This is the conditional circumstance of all cells when multicellular eukaryotic organisms are properly ascertained as holobionts [11].

Within a standard evolutionary perspective, tumor progression is regarded as intrinsic to the relative fitness of cancer cells based upon stochastic tumor mutations, of which aneuploidy is one variety. The subsequent ecological effects are varied and include both competition and mutualistic interchanges between co-evolving clones in and around a neoplasm. It has been typically assumed that cancer is a reiterative process that proceeds through clonal proliferation and genetic diversity along an adaptive landscape within tissue environments according to Darwinian precepts [39]. Within this context, Greaves and Maley [39] indicate that each cancer is unique to any individual, variable over time and composed of cellular lineages with distinct genetic mutations and phenotypes that can represent sub-clonal cancers occupying distinct or overlapping tissue habitats. Moreover, such genetic diversity permits exploitation of tissue ecologies through increased proliferation, migration and tissue invasion. Crucially, it is acknowledged that the manner in which this occurs is best understood as “part and parcel of normal developmental, physiological and repair processes” [39, p. 306] in which aneuploidy is one of many contributory factors. Although the direct association is accepted, the exact role of aneuploidy in cancer cellular dynamics and its relationship to chromosomal instability (CIN) is still uncertain. That CIN could itself be the source of genomic instability is one consideration [40].

Any view of evolution with self-referential cognition at its center represents a comprehensive alternative to standard NeoDarwinism. When cancer proliferation is considered in that frame, cognition based evolution (CBE) offers explanatory factors beyond selection and stochastic processes. In this construct, evolutionary development is continuously based upon communication between self-referential constituencies that yields non-random problem-solving at the cellular level reiterated at every scope and scale [10, 41].

These cellular outcomes proceed through natural cellular engineering. This process is based upon those cellular mechanisms through which the innate cells of any organism and its co-existent microbial fraction compete, collaborate and cooperate in complex ecologies that constitute a holobionic entirety. At all times, cells are acting to support their own self-referential optimized homeostatic status [11, 42]. These actions are properly considered cognitive, but it deserves emphasis that this is limited to basal discrimination of status and is not comparable to the decision matrices by which biological entities with higher consciousness settle ambiguities.

The manner in which natural cellular engineering proceeds is best understood through the biological concepts of niche construction and stigmergy. In conventional Darwinian terms, niche construction is conceived as the means by which macroorganisms modify their own environment. Indirectly then, they influence and modify the environment of others. It is believed that in this way, organisms deal with selection pressures that act upon themselves and any future generations [43]. Since all cells are communicative, flexible and can adapt according to their limits, the principles of niche construction can be properly applied to intracellular and cellular dynamics [14, 44]. Placed in a self-referential frame at the cellular level, niche construction becomes a directing process towards common cellular aims and cooperative purposes operating at that scope and scale and can proceed without any external direction. Instead, cells enact ecologies along terms that can be considered engineering but is simply an iterative process that is consistently directed towards maintaining individual self-referential homeostasis best achieved through collective action. This requires no external engineer as it is based on local reinforcement and reciprocating cellular interactions, and can be considered similar to those impulses that propel the growth of human settlements and cities [11, 18].

Stigmergy is a mechanism of indirect coordination between agents and their actions within an environment that has been typically considered within a standard Darwinian narrative [45]. The stigmergic principle is that any trace left in the environment by the action of an agent stimulates the performance of a next action, by the same or a different agent. Any living entity whose goal is to maintain self-identity by sustaining a preferred homeostatic boundary condition would satisfy that requirement. Although stigmergy has been typically regarded in the macro frame, such as termite mounds, it has been identified as fully operative in the swarming behaviors of unicellular organisms such as myxobacteria in which the stigmergic cues are regarded as chemical interactions between cells [46]. Eukaryotic cells have an entire panoply of communicative and cooperative mechanisms [8, 11]. Since the individual cellular participants can have independent goals in any mixed cellular ecology, there is a natural division of labor. The variety of these participants working together through stigmergic paths builds complexity, in sequence or in parallel, based on a continuous stream of information from within any niche or those aspects of any conjoined information field that is shared with other niches. It is the same whether elaborating an ant or a bacterial colony [47]. Further yet, it is just such self-same cellular capacities and processes that leads to holobionts as the end-point of eukaryotic evolution. At every scope and scale, cells are connected together in networks that jointly seek to problem-solve and adapt to changing environmental circumstances.

Importantly too, as a condition of multicellular eukaryotic life, development remains anchored within cellular terms to the fundamental unicellular form despite any outward macro form [44]. Evolutionary development and the architecture of collaborative multicellular ecologies is therefore continually based on a set of reiterating First Principles of physiology which represent the range of homeostatic responses that cooperating cells can maintain to cope with environmental change, and have been sustained since the inception of the unicellular form of life [13, 44].

### Beyond NeoDarwinism

There are a range of far-reaching consequences that spill from any altered evolutionary narrative in which cellular imperatives dominate that apply to neoplasia. Our modern reappraisal of the importance of epigenetic factors in evolutionary development is a significant component of this differing perspective. The Lamarckian inheritance of acquired characteristics is now acknowledged as an essential aspect of evolutionary development [48–51]. When the circumstances of eukaryotic life are re-examined with cognition at its base, the survival of any organism is information dependent. Although it is our natural instinct to award primacy to our macroscopic appearance through which nearly all Darwinian scrutiny appertains, it is information that actually matters most in evolution [10]. Information underpins self-recognition and maintains homeostasis at every scope and scale. As opposed to any macro manifestation, when the cellular form is deemed central to all eukaryotic multicellular organisms, the unicellular state through which all multicellular eukaryotes must recapitulate can be reappraised. It has been asserted that the unicellular state is the perpetual epicenter of life [44]. That reason is centered on the quality of information that is required to sustain the macro whole. All multicellular eukaryotes experience an obligatory return to the unicellular phase. Through meiosis and the unicellular zygotic phase that then follows, there is a necessary adjudication of the epigenetic marks that are acquired in the macroscopic form and are hallmarks of biological information. These are thereafter adjusted in subsequent developmental stages. At each stage, the quality and utility of the information available to the participating cells is being assessed. Therefore, evolutionary development does not merely extend forward from unicellular roots, but remains anchored to those fundamental linkages in perpetuity [52] as the stage in which the eukaryotic entity recenters the information that it will use for its next macro elaboration. In consequence, evolutionary development is not just a closed path by which contemporary organisms have achieved current biological form and function. Instead, some of the evolutionary mainstays that have been part of past experience remain available through re-connections that perpetually recapitulate through the unicellular phase. The unicellular phase is thereby always in continuous contact with its evolutionary past in a manner that the macro differentiated elaboration is not. Therefore, in a cellular world, prior solutions can be re-explored when the necessity arises. This becomes a continuous evolutionary toolkit based on First Principles of physiology which have developed in direct response to environmental stresses [12, 13]. In this manner, evolutionary fluidity becomes one means by which cells can solve homeostatic problems that relate to any current agitating external milieu.

When the eukaryotic unicellular zygotic form is properly understood in its disciplinary role in sorting epiphenomena, and further yet, it is appreciated that it does so as a self-referential entity purposed towards its perpetuation, then phenotype can be reconsidered as being in service to that unicellular stage. Phenotype is the means by which the unicell explores the environment. It does so through the accumulation of epigenetic marks acquired in its macro phase which are then adjudicated in the unicellular phase [53]. Therefore, it can be understood that phenotype is a tool directed towards the perpetuation of the unicellular form [44, 53]. The unicell requires the agency of phenotype as its means of gathering information in its attempt to remain in equipoise with a variable external environment. Although information based on epigenetic impacts is effectively gleaned in this manner, it can also be appreciated that information of this type is a consistent source of ambiguity and stress for all living entities. In living systems, information is inherently ambiguous and best considered as a spectrum of superimposed Bohmian implicates and potential explicates that underscores the essence of biological systems [54]. Bohm believed that reality is a continuous stream of overlapping implicates and explicates that are evaluated through our senses but often mislead us. At all times, our condition includes broad sets of subjective implicates of which we are often unaware.

The manner in which the eukaryotic unicellular phase, or any cell for that matter, settles these superimposed possibilities represents its problem-solving capacity. Every cellular unit is a coherent and discrete cognitive entity, but importantly, in any information network, noise must be regulated. Absent such a mechanism, chaos is ultimately unavoidable. Therefore, there must be a means towards either the expression of epigenetic marks or their down-regulation towards biological resolution. It is presented that this process is best understood as the continuous settling of the quantum superimposition of possibilities stirred by ambiguous epiphenomena in cellular terms [10]. Such quantum phenomena are functions of the information fields that all organisms occupy based upon all the sources of input to any living entity [10]. It does so by utilizing the full range of information to which it is connected, as its relevant information field that constitutes all actual and potential knowables as a living entity. In this manner, any self-referential agency can settle coherences of integrated information that can be expressed as explicit biological outcomes [10, 55]. The zygotic unicell gains this integrated information about the external environment through its transient elaborating context through macro phenotypes, but utilizes the information that returns according to the proscriptions of its own self-referential state. Through this exclusive path, the unicell becomes both privileged cellular observer and participant by fundamentally retaining its ancient roots as a primordial cellular entity, but consistently acquiring information through an endless stream of epiphenomena [8].

With this background, CBE can be understood as a consequence of cellular imperatives in which self-referential cells sustain homeostatic preferences in the face of a stream of stresses as biological ambiguities. In essence, each cell, and its network, appraises its information field and reacts as it can. In turn, that cell constitutes its own information field to other self-referential entities. Phenotype is its product and is enacted through natural cellular engineering mechanisms. It proceeds forward from unicellular particulars, but remains in contact with its evolutionary roots through its obligatory recapitulation through the unicellular phase. Such natural engineering processes can thereby be seen as a continuum from the origin of life forward as a consensual application of cellular purposes which are adjusted to contemporary circumstances, but sustain fidelity to their original toolkit. Since natural selection follows phenotype, selection can be properly appraised as a post-facto filtering agency. Therefore, phenotype is a product of both self-referential problem solving in its first instance, and filtering selection of the residue.

Physiology sustains cellular self-awareness within CBE. Physiological processes are directed towards maintaining homeostatic preference amid changing environmental circumstances and macroorganismal stresses. Still, physiology must proceed at the cellular level to achieve its macroscopic effects. It can therefore be seen as another collective product of cellular engineering along stigmeric pathways that is in constant reciprocation with the self-referential requirements of the cell.

As an important derivative of an evolutionary system based upon information quality and transfer among self-referential entities rather than reproductive frequency, genes and also oncogenes become informational tools. Genes serve self-referential cognition as a crucial component of its information system. In this manner, genetic transfer becomes communication in information space with its own forms of ambiguities, implicates and future explicates. This perspective supports the emerging recognition that the long-standing paradigm of cancer origination from isolated genetic mutations must be supplanted by an enlarged one in which the concerted action of dysregulated oncoproteins overwhelm cellular defenses against cancer proliferation. Towards that end, algorithms such as Virtual Inference of Protein Activity by Enriched Regulon Analysis (VIPER) are being used for the assessment of aberrant oncogene expression [56]. These investigations are not only being used to accurately infer abnormal proteins from known genetic mutations, but for the evaluation of tumors that have aberrant oncoproteins despite the lack of mutations. Cancer is thereby being understood as an integration of proteomics and transcriptomics in which the relationship between genetic mutation and cancer is indirect. Therefore, a background model that conceptualizes cancer dynamics in terms of a self-referential cellular problem-solving agency is inherently more flexible than the prior Darwinian/mutation selection one.

Of particular importance in CBE is the role of immunological reactions in supporting and reinforcing self-recognition juxtaposed against a continuous stream of external environmental stresses and epiphenomena [11]. Cognitive self-awareness is a function of those immunological means that enforce the identification of ‘self’’ from ‘other’ within an active biological frame. Consequently, immunological reactions support and reinforce self-recognition in apposition to a continuous stream of external environmental stresses and epiphenomena. This dynamic has been continuous from life’s inception and is a foundational aspect of evolutionary development [10, 11]. Therefore, in all circumstances in which tissue ecologies have a broad mixture of self-referential constituencies, immunological factors rule.

There is a singular dynamic that underlies CBE as an alternative to standard NeoDarwinian selection. All aspects of life are directed towards communication and organized problem solving by cognitive entities [57]. This is the exact context of understanding a self-referential cellular world. At every scope and scale, all living entities and cells use information to resolve environmental ambiguities into explicate self-referential biological solutions. Further yet, when self-referential entities purpose the settlement of biological ambiguities against homeostatic constraints, then solutions represent deterministic creativity as opposed to mere selection and stochastic variables. In any self-referential cellular context, creative solutions are enacted across linked networking constituencies to reach consensual cellular solutions to environmental stresses. This is the process by which separable living entities become holobionts. The center of all such activity is information transfer. When enacted through biological organisms as communication among self-aware participants, cellular responses ultimately become creative biological expressions to resolve environmental stresses.

## Cancer evolutionary homologies in a post-Darwinian frame

### Cancer as a self-referential agency

All eukaryotic cells are self-referential. Since it has been long recognized that cancer cells represent distinct cell lineages separable from the normative genomic structure [2], they are a unique self-referential cellular population. All cells of any type can be productively considered as sender/receiver units that are largely defined as a summation of all their communications [57]. However, any cognitive entity communicates through its connection to information space in the context of the changing information field that both directly and indirectly confronts it. In turn, that same entity is its own variable information field towards the outward environment [11]. As derivatives then, cancer cells deal with information fields and consequent ambiguities within their own specific context in the same manner as other cells. For example, T cells function as a self-referential agency and as a sensory cell comparable to any in the nervous system [58]. The capacity of that response is so broad that T cells have been likened to sensory organs.

Since all cells are self-referential and utilize information space to assess status within tissue ecologies, cancer cells must also do so according to their own individual homeostatic requisites. Given that the abnormal cancer karyotype is separate from the background normative genomic structure of the other innate cellular constituents within any local tissue ecology, any cancer cell could be surmised to have its own discrete self-referential use of information space, and would then use it to shape its actions within any localized tissue ecology. In this manner, it is not differing from any microbial participant with its own ‘self’ in any localized tissue environment. By implication via their differing ‘selves’, cancer cells would analogously have the capacity to act as distinct problem solving agencies. However, their context is unique. They are specific and separable self-aware participants in a tissue ecology, but also have exclusively originated from within the progenitor cellular milieu. Therefore, cancer cells would be expected to occupy privileged observer/participant status compared to other innate cells.

Any tissue ecology represents its own collective information space. All cells participate within that field and any cancer lineage would depend on it as distributed across any local tissue microenvironment in which cancer exists. Therefore, it is an expected outcome that cancer cell lineages, arising from the background cellular matrix, at least at first, are active participants in those initiating tissue ecologies, with levels of collaboration, cooperation, and competition in reciprocation with other ecological participants [59, 60]. However, as its own self-referential entity, and further yet as a consequence of its privileged participant/observer status as apart from normal cells, cancer stem cells become their own epicenters from which any clonal lineage might arise for the adjudication of epiphenomena. From this exceptional state, the cancer stem cell functions in a similar manner to the eukaryotic master unicell bearing some resemblances to the recapitulating zygotic form in its flexible adaptation to epiphenomena.

### Ambiguous immune status

Any cancer stem cell is instantiated as an independently self-aware agency. Yet, it still arises from a cellular origin derivative of the normative cellular genomic state [61]. Hence, at least at first, cancer is not necessarily appraised by other cells as a foreign entity within the local tissue ecology since it shares features of the common information field that influences all other cells in its local microenvironment. Therefore, despite its independent status, it successfully evades typical immunological mechanisms by which any foreign ‘self’ would ordinarily be identified and is instead assessed as consonant with the general homeostatic status of the localized tissue ecology. Importantly, this permits the new clonal lineage to be an active participant in localized tissue ecologies, enabling cellular reciprocation and the trading of resources within the ecology which might ultimately proceed beyond the typical control mechanisms [62]. It thereby establishes its foothold. The cancer lineage has not merely evaded the immune system, but is actually recognized as a co-aligned entity, and is permitted to share in ecological resources. Unlike a bacterial, fungal, or even any typical viral player, an uncanny parasite of this sort might be nearly or entirely shielded on an immunological basis. Therefore, even as a foreign agency with respect to its self-referential purposes, cancer is initially permitted to proliferate with little opposition, and can actually recruit ecological resources for its own exclusive niche construction. The shared origins of this initially co-aligned cell, no matter the trigger that initiates CIN or chromosomal mis-segregation, permits an overlapping connection to the information space upon which the innate cells of the cellular ecology depend. That serves as its means towards further development of its clonal cellular lineage apart from the normal cell background within the shared context of the inherent ambiguities intrinsic to all information fields. Therefore, ambiguous information is the opportunity that cancer stem cells employ. Cancer’s privilege is its start as an equivocal immunological entity vis-a-vis the entire tissue ecology in which it first arises as an obligatory participant in information space. Its communicative status is at a sufficient level of ambiguity that natural mechanisms for sustaining cells are licensed as part of its repertoire. In this manner, cancer is permitted to maintain its own self-referential identity within its environment despite robust mechanisms to resist intruders.

### Aneuploidy and phenotype

Aneuploidy is widely understood to be a common characteristic of cancer [63]. Yet, it still remains uncertain whether aneuploidy is the cause or consequence of malignant transformation [64]. Despite that uncertainty, it is acknowledged that aneuploidy is a consistent feature of cancer cells associated with chromosomal mis-segregation and CIN with defects centered within mitosis, cell cycle surveillance, the centrosome cycle and the spindle apparatus [65, 66].

It is clear that normal diploid cells with the same genomic characterization have different patterns of genetic expression that are context specific. Such fixed patterns of expression permit those cells to define different organs and tissues. Nicholson and Crimini [36] indicate that similar tissue-specific patterns govern the manner in which aneuploidy expresses. Therefore, the site and specific tissue of origin are important in determining karyotypic patterns of cancer cell so that similar aneusomic chromosomes can demonstrate different patterns of gene expression in differing tissue contexts. Therefore, it can be assumed that aneuploidy has an ambiguous status within any tissue ecology. In consequence, aneusomic cell lineages could result in cells with differing immunological imprints.

In standard Darwinian terms, aneuploidy has been typified as altered selection-dependent phenotypic expression directed towards differential fitness in an adaptive landscape [67]. However, its adaptive potential relates to context. In specialized circumstances, it can serve the organism to its betterment, such as by conferring forms of resistance to stress based on altered genetic expression in a tissue-specific manner [36]. Consequently, aneuploidy can be alternatively viewed as a mechanistic toolkit for cells to solve problems in ambiguous circumstances. In some situations, the resulting phenotypic expression provides a survival advantage. In others, the aneuploid cell pattern expresses differently and becomes the basis for neoplasia through enhancement of mis-segregation and a further proliferation of differing karyotes. However, in either set of circumstances, upon inception, that initial aneuploid cell is a differing self-referential entity with its own distinct and indeterminate potentials. In each instance, it becomes an alternative phenotype directed towards exploration of any outward environment seeking solutions to its own assessment of environmental stress that is, perforce, separated from normal cells. Dependent on site of origin and context, aneuploid cells that become cancer might be initially tolerated as part of the problem-solving potential of cellular ecologies precisely due to its dual nature and consequent ambiguous status.

Although aneuploidy may itself be a progenitor of cancer, it has been widely considered as possible indirect agency in CIN and chromosomal mis-segregation [68]. The connections are not necessarily absolute. It is generally considered that it is only when genomic instability supervenes that the activation of the cancer phenotype begins [69]. However, this apparent inconsistency can be productively resolved by regarding any cell with any variant karyotype as its own self-referential agency that exists within the context of an extensive cellular ecology in which it must find succor. This is the same milieu that all cells experience. However, when subjected to a triggering event (spontaneous mutation, dysregulatory oncoproteins, or epigenetic impact such as viral/retroviral incursion, a carcinogen, or a mutagen), an ordinarily limited and constrained biological mechanism becomes undisciplined within the cellular matrix within which it arises. Importantly, when such an event supervenes, a new cellular agency is effected, aneuploid or not, with its own self-referential center and its own proscriptions, purposes, and solutions to stresses.

It is known that cancer proliferation manifesting through chromosomal instability rapidly expresses a range of phenotypes. Certainly, cancer is routinely considered on the basis of these numerous, variant cellular phenotypes [70]. Since all cells can judge their environment, it can be asserted that cancer stem cells capable of initiating a novel primary cellular lineage might have such a capacity and meet it with greater flexibility. In cellular terms, this might be productively considered as a progenitor cell with exceptional participant/observer status within the information space of localized and even distant cellular ecologies. It can be offered that this capacity constitutes the atypical flexibility of the cancer genome and its transcriptional/proteomic output. As such, a progenitor stem cell and those neoplastic cells that originate from it become an agency of a discrete and idiosyncratic phenotype. In our biologic system, phenotype is an agency of exploration of the outward environment. Whether expressed through macroorganisms or cellular lineages, it is always in service to the dominant unicellular form through which it must recapitulate [44]. In like manner then, CIN as expressed through individual cell lines can be viewed as alternative forms of phenotypic variation that permit the initiating stem cell lineage to explore its own greater environment. In the case of cancer, this includes both local and distant tissue ecologies. Therefore, for neoplastic stem cells, CIN is its tool in information space towards its furtherance of neoplastic cellular ‘self’ as only it can interpret.

Cancer has been considered a stem cell-based disease [71]. CIN permits the neoplastic stem cell to explore its own microenvironment within tissue ecologies according to the differing phenotypes that proliferate. Each proliferative outpost becomes a co-respondent clonal constituency of the initiating stem cell, remains in communication with it, and permits continuity and adjustment for the neoplastic stem cell. In this sense then, the relationship between the zygotic unicell and its allegiances and its evolutionary path as expressed through the eukaryotic macro form is mirrored by the cancer stem cell and its elaborating clonal lineages. Cancer should thereby be considered an atypical expression of phenotype that is its own reiteration of identifiable evolutionary patterns. The latency between instantiation of cancer as aneuploidy or CIN in any local tissue ecology and its ultimate aggressive intention, is a function of its self-referential requirements as a clonal initiator, the limits of its problem solving capacity and its capacity as an initially covert immunoresponsive ecological participant. By this means, as a self-referential agency, cancer proliferation becomes an issue of neoplastic cells exploring their environment, as they construe it, towards the furtherance of ‘self’. It is enabled by iterative feedback from its clonal progeny that permits proliferation in successive rounds. This is self-referential neoplastic cellular engineering as opposed to simple selection. There are, then, two further implications to the viewpoint of neoplastic cells as self-referential agencies. The success of neoplasia is not merely a function of raw reproductive frequency, and further, any such cells must still retain some kinship to their own unicellular origins despite its aberrant phenotypic expression.

### Cancer cell engineering

It is known that normal tissue homeostasis is maintained by dynamic interactions between cells. As cancer cells arise, there are reciprocal interactions between these neoplastic cells, any adjacent normal cells such as stroma and endothelium, and their respective microenvironments [72]. Therefore, cancer cells, though differing in ‘self’, have the ability to retain the same cellular capacities that are exhibited by all other cells in any tissue ecology. All cells maintain self-identity by sustaining preferred homeostatic boundary conditions. It can be assumed that neoplastic cells would be capable of utilizing the same panoply of faculties that are conferred to any cellular constituency within any tissue ecology. Therefore, just as all self-referential cellular entities engage in active niche construction, cancer cells do the same.

Bergman and Gligorijevic [73] assert that processes contingent on the proliferation of cancer and eventual cancer metastasis include construction of niches. This mechanism is directed towards their perpetuation, but also explores pathways to transport neoplastic cells to new environments. It is the consistent nature of macroscopic organisms when engaged in niche construction to actively explore for new niches, and cancer cells as self-referential entities would be expected to conform. However, such lineages engage in niche construction from a position of particular cellular privilege based upon their separable karyotypic ‘self’ and the flexibility inherent in stem cells as opposed to fully differentiated ones.

It is known that neoplastic cell lineages have the capacity for their own expression of ‘immortality’ [74]. Therefore, it can be assumed that cancer engages in its own exclusive form of niche construction within its own discrete boundaries as a self-referential entity towards that end-point and utilizes aneuploidy or CIN as tools. That a pluripotent cell, such as a cancer stem cell, should be viewed as an independent cognitive entity capable of its own specialized niche construction is not merely conjecture. There is medical proof of the validity of that assertion. Stem cell transplantation for medical treatment has been shown to be associated with tumorigenic potential. A recent case of a glioproliferative lesion of the spinal cord after intrathecal injection of mesenchymal, embryonic and fetal neural stem cells illustrates that cellular features that overlap neoplasia can arise de novo from transplanted stem cells [75]. Thus, there is discrete evidence of pluripotent cells acting as an independent, self-directed problem-solving agency within tissue ecologies that are distinctly foreign to their organism of origin. In this way, neoplastic stem cells bear similarities to other independent organisms as is separately exemplified by the entire microbial sphere.

Among those tools that cells employ in natural cellular engineering, stigmergy is an important means. Certainly, it would be expected that cancer must also utilize this basic biological mechanism that is common to all cells. However, when placed in the context of reciprocation, competition, and traded resources within mixed cellular ecologies, any privileged participant might gain an out-sized advantage. In normal circumstances, constituencies participate together within boundaries, and from this mix, despite differences, complexity builds. As a process, its application is based upon an existing information stream through a division of labor. The unique capacities of any neoplasm as a different and initially ambiguous ‘self’ within any typical ecological mix empower cancer to elude typical cellular boundaries and limitations. Since any information among constituents in any tissue ecology is both direct and indirect, and is disseminated under conditions in which neither sender nor receiver is necessarily clear-cut, conflicts are initially diminished. At least within a first iteration within mixed cellular ecologies, cancer and all other constituents mutually edge towards consensual outcomes, each attempting to sustain their own limits. As cancer cells engage in these typical cell processes, common to all cells, they co-opt ‘normal’ cellular resources and do so with an effectiveness that relates to their unique participant/observer status and then, too, their own individual needs that are differing from normal cells.

At any first initiation of a neoplasm, cellular synergy is directed towards the seeming interests of the conjoined cellular constituencies within localized tissue ecologies. This is an immunological deception based upon a biologic system in which aneuploidy is not necessarily a negative within cellular ecologies. By this means, neoplasia can be modeled as a form of particularly effective parasitic process masquerading as a cellular ecological participant [76]. When cancer is finally well along its stream of phenotypic exploration in response to any localized microenvironment, other participants in local tissue ecologies can collectively organize to engage in countervailing defensive niche construction. This then yields the microscopic and macroscopic manifestations at local boundaries of cellular and inflammatory reaction to neoplasia [77, 78].

### Reverse evolution in cancer

Any stem cell that is the progenitor of a new lineage occupies a privileged status. Cancer, as a self-referential state independent of the normative cellular ‘self’, is empowered towards the exploration of its information space. It will thereby collapse the superimposition of latent variables into those that are best equipped for its non-random survival and perpetuation. It actualizes this by taking current epigenetic marks and transforming them simultaneously into both historical experience and latent future heritable explicates, just as in the circumstance of the zygotic unicell. By this means, the next proliferated form of neoplasia as phenotype is both a ‘present’ and a forecast of future trends. It judges this according to the broad parameters of its homeostatic limits as an independent self-referential entity and further, through its simultaneous connection to the genomic circumstances from which it arose.

 It can be expected that any cancer lineage that is its own self-referential entity with a capacity to adjudicate epiphenomena can then be expected to have potential connections to the types of tools available to the unicellular state. This is actualized through its own individualized use of its unique cellular/genomic endowment from which its ‘self’ arises. Indeed, it is known that within tumor tissues, there are tumor specific stem cells that appear to exist within a progenitor state of development [79]. The concept that cancer is in some way connected to its ancient roots or even the origin of life is not new [80, 81]. Fernandes et al. [81] have proposed that neoplasia is exceptionally connected to the prokaryotic homolog toolbox. The successful proliferation and longevity of cancers are sustained by several factors: high glycolysis, chemoresistance and radioresistance. However, these are all shared metabolic features of many cell types that include malignancy and the unicellular sphere. Such traits arose early in evolution and have been sustained among prokaryotes. This backward connection towards its primordial toolkit enables the aggressive proliferation of cancer cell lineages. It does so at the expense of other ecological partners through the successful re-acquisition of unicellular behaviors which are believed to be due to the over expression of viral or prokaryotic homologs. It has also been suggested that this backwardization is triggered by the dysregulation of mitochondria [82]. This process is hypothesized to be due to cumulative oxidative damage to mitochondrial and nuclear DNA. Through this process, the phenotype of a previously differentiated cell reverts to the phenotype of a facultative anaerobic, heterotrophic cell optimized for survival and proliferation in primordial hypoxic environments. To effect its survival strategy, it has been proposed that the atypical cancer phenotype attempts to mirror the phenotype of the Last Eukaryotic Common Ancestor (LECA) such that cancer represents a recapitulation of the evolution of the eukaryotic cell from fully differentiated cells to their LECA form [82]. Once these obscured traits are expressed by the proteome of a tumor, an explicit advantage is conferred, particularly since these same originating traits are those that granted survival advantage to prokaryotes in harsh environments. Thus, the specific toolkit of cancer is its ability to slide along the evolutionary narrative of its ‘self’ to achieve unusual flexibility in its encounters with epiphenomena. This special flexibility becomes a part of its privileged participant/observer status among other constituencies in local or distant tissue ecologies as it seeks its preferred status.

In this way, chromosomal instability is a means of rapid expression of a range of phenotypes and pleiotropic/epistatic flexibility enabled through its ability to singularly reconnect with its evolutionary past [12]. It is known that cancer lineages have the ability to avoid the normal checkpoints of cellular regulation to empower longevity [83, 84]. This same exceptional facility permits successive rounds of under-regulated genetic variation and the backtracking of the cell towards more primitive pluripotential forms. It is through this means that cancer emerges as a highly skillful and privileged tissue ecological participant. The effect of this advantage is hyperadaptibility [85]. Cancer cells explore the range of implicates vis-a-vis epiphenomena faster than normal cells by particularly potent connections with prior evolutionary tools permitting problem-solving, de-differentiation, and pluripotency with respect to a competitive or cooperative stance with normal companion cells.

The impulse for reverse evolutionary backtracking is cellular responsiveness to epiphenomena. Cancer is not the only aspect of the eukaryotic cellular domain that can reverse engineer. In all multicellular organisms, some cells are granted this capacity. For example, tyrosinemia-induced stress in the mouse liver can be overcome by the induction of aneuploid hepatocytes lacking chromosome 16. As such, reverse evolution can be seen as a complex capacity that exists within all organisms rather than a pathologic feature [36]. The difference lies within the limits of flexibility of that reach.

Nor is such a capacity confined to the cellular sphere. Research indicates that a primitive mosasauroid from the Middle Turonian (*Dallasaurus turneri*) evolved from a terrestrial animal to a fully aquatic one [86]. The independent evolution of flipper like limbs from terrestrial ones appears to have happened at least twice [87]. Further yet, Guex [88] has demonstrated a relationship between retrograde evolution and extinction events, noting that the types of observable regressive changes occur in like manner between protists and unicellular forms and metazoans across time.

For all eukaryotic multicellular organisms, adjudication of epigenetic impacts is effected via an obligatory recapitulation through the zygotic unicell. This is preceded by meiosis and then followed by a regulatory embryological phase. Neoplasia utilizes genomic instability or lability as its means, skewing from normal cells based upon its own exclusive adjudication of the impacts of epiphenomena as it reaches towards its own idiosyncratic homeostatic needs. Reverse evolution constitutes one of its tools. In cognition based evolutionary terms, tumor pluripotency means the deployment of a wider range of implicates, and the differential use of information space compared to normal cells.

Importantly within any self-referential construct, unless information is expressly directed and received, it becomes a primary form of biological ambiguity. It can be suggested, then, that the capacity for neoplasia to connect to both its present and past empowers its ability to more swiftly settle ambiguities than other competitors or collaborators in its ecological milieu. This hyper-adaptable environmental responsiveness has been emphasized [89]. In essence, cancer out-competes through better information quality based upon its rapid and flexible exploration of its ecological milieu, which, if properly considered, can be appreciated as its information space. Although tumor evolution has been typically seen as genomic instability in a frame of differential fitness responses [90], it is instead asserted that the success of cancer is the combined function of self-referential status, information quality, information exchange with ecological participants, and a cellular dynamic that permits the backtracking of the typical evolutionary direction that proceeds via tumor exaptations of a primordial evolutionary toolkit that has always extended forward from unicellular roots. When placed in the context of an independent neoplastic stem cell and its consequent lineage, this can be understood as a flexible reconnection with adaptive solutions from the past. In consequence, a significant change in our frame of reference regarding neoplasia and its genomic stance is necessitated. In typical terms, a genome is considered a read-write mechanism akin to lines of code [91]. However, in the context in which cells are self-referential problem-solving agencies, genes are flexible tools that have employed to empower solutions to stresses that extend beyond any model that resembles computer software. Instead, any genome, including any neoplastic one, is a flexible response system with an inherent memory of past adaptive solutions that can contribute to self-referential cellular survival and proliferation.

### Further implications of CBE

When cancer is placed in a self-referential frame, there are several derivative aspects that explain cancer dynamics. Within any cognition-based system, it is not necessary that all cancers exhibit purely genetic stochastic variation. Instead, the purposeful utilization of genes and other cellular agencies as tools permits the cancer lineage to solve environmental problems and relieve consequent environmental stresses. The concept that genes are passive code is no longer tenable. Instead, it is now known that they are flexible implements capable of a broad range of expression based on a variety of influences that have only recently been considered [92]. Therefore, genes and genomes are flexible tools in service to cognitive agencies that purpose phenotype as an output towards the exploration of the external environment [53]. If the entity that purposes phenotype is acknowledged as self-referential, then the effects of CIN should no longer be viewed as only stochastic. Instead, from whatever cause, CIN is directed towards solutions to environmental stresses when it arises, which would then account for the conditional variant expression of aneuploidy in cellular contexts. Indeed, there is research evidence for this proposal. Non-random mechanisms have been shown to exist for neoplastic chromosomal rearrangements in adenoid cystic carcinoma of the salivary glands [93]. Other non-random aspects of oncogenesis, such as aspects of viral integration of the HPV genome in cervical cancer, have also been documented [94].

The absence of apoptosis and the immortality of cancer clonal lineages provides a means for cancer to occupy a position among constituent cells in any tissue ecology as a privileged observer/participant. As noted, from that position, CIN and phenotype effectively become effective tools of a purposed self-referential dynamic. From this, there is a further implication. It has been advanced that the unicell should be considered the first example of niche construction [14]. If so, it then follows that cancer stem cells are themselves examples of niche construction based upon intracellular tools and CIN. Furthermore, as cancer stem cells uses phenotype to explore its local tissue environment, cells of the same clonal lineage that circulate to differing tissues are pathfinders towards future niches. It should be expected that these exploratory cells remain in communication with their neoplastic clonal lineages at their site of origination and well as others dispersed throughout the entire macroorganism. In this manner, neoplasia gains a toehold at distant sites in non-random manner that always remains in concert with its own defined ‘self’.

## Discussion

Cancer cells are cognitive agencies that deal with ambiguous information to seek solutions within cellular environments. Therefore, cancer is a form of cognitive entanglement with other cellular constituents within its milieu. It follows the proscriptions of cellular life or the cellular skills that might be utilized including cooperation and competition, active trading of resources, and transfers of information that are similar to any other constituent within any mixed cellular ecology. Yet, it interacts according to its own ‘self’ and utilizes specialized skills. These include the rapid exploration of its environment through flexible phenotypic shifts, unusual longevity, re-connection with its evolutionary path, and its singular capacity to elude the normal cellular checkpoints that constitute the regular orderliness of cellular life. In part, it achieves dominance vis-a-vis other constituents in any tissue ecology through flexible regressive evolution via tumor exaptations of a toolkit that extends to its unicellular roots. Therefore, cancer is not merely a stochastic Darwinian exigency but an active, propulsive, agitating, self-directed and self-referential agency although limited by its level of basal cognition. Yet, cancer must conform to physical limits just as all cell do. All cells attempt to sustain states of preferential homeostasis through purposeful actions according to their scope and scale. As a necessary derivative manifestation of our physical system, this activity is directed towards the minimization of variational free energy and the suppression of surprise (unpredictable outcomes) [95]. Therefore, cancer would be assumed to act in like manner within its own self-referential limits.

The concept that cancer cells can have a significantly separate identify from their background cellular ecology of origin is not new. Others have even suggested that cancer cells are an expression of speciation [76, 96]. In typical Darwinian terms, the slow kinetics of carcinogenesis has been explained by the low probability of random chromosome re-assortment that could yield an aggressive proliferating phenotype. However, beyond any academic consideration of the accuracy of considering differing cancer cell lineages as separate species with all its attendant definitional problems, the issue can be more directly addressed by acknowledging that cancer represents a differing self-referential agency within any localized microenvironment. Its actions thereby extend beyond random occurrences. CBE proposes that cancer cells have a purposeful connection with their information space and access it according to their separable faculties in service to problem-solving within their context. This becomes a self-referential feedback loop whose effectiveness can be considered an example of recursive causality. This concept represents the idea that every biological effect on an organism feeds back to its own cause. Noting the continuing problem connecting genes and forms solely through adaptation and selection, and the variable impacts of epigenetic impacts and transcriptional regulation, Haslberger et al. [97] suggested that a framework of recursive causality would provide a robust link between molecular biology, cognition science and systems theory. Cancer becomes an example of the extent of that feedback to its roots and its unique linkage to its contemporary circumstances and its own information space.

Certainly, whenever the cognitive aspects of cells are adduced, there is a tendency to be skeptical of the range of behaviors that could be attributed to cellular entities. However, there is no need for a brain to make complex decisions. Reid et al. [98] studied Physarum *polycephalum*, a unicellular slime mold that can grow to exceptional size. Experiments demonstrated a surprising range of complex behaviors including solving complex mazes, making exploration–exploitation trade-offs, remembering past locations, engineering and constructing sophisticated transport mechanisms and anticipating periodic events. These faculties reinforce that information processing and cognition are widely distributed across all living things through a shared ancient ancestry. Furthermore, it has been shown that cells can implement complex computations based upon a stream of epiphenomena [34]. Such computations are rough forms of both analog and digital signal processing that cells use to construct complex developmental programs, context-dependent behaviors and sustain homeostatic balance.

Cells of all kinds, and cancer cells too, have substantial problem-solving capacity. As a result of CIN and chromosomal mis-segregation, whatever their explicit causes, all resulting clonal lineages are properly considered as agencies towards separate cancer phenotypes. In such circumstances, then, each clonal iteration must be regarded as a differing ‘self’. Each is separated from prior clones in its actual memories, deployment of that memory, information field, and connections with information space. Its privileges are a product of its malleability through the absence of normal cellular checkpoints with a concomitant ability to slide backwards with some flexibility along an evolutionary scale since it maintains a connection to its evolutionary problem-solving capacity. Therefore, each of these new phenotypes, as separable iterations of ‘self’, can explore the tumor microenvironment, and others beyond, in its own manner by encountering problems and attempting to solve them. Neoplastic latency can be regarded then as a balance of forces between cancer phenotypic ‘selves’ and the tumor ecology [99]. In any tissue environment, cancer ‘self’ is in juxtaposition to other self-referential constituencies that might be competitive or cooperative, each with its own cellular aims and ecological proclivities. In each circumstance, that balance is positioned between the countervailing problem-solving limits of the opposing players.

In a cognition-based framework, the issues of cancer mutation, clonal lineage, and cell–cell fusion can be reappraised. There has been wide acceptance of a two-stage model for cancer progression [100]. It has been proposed that mutations and aneuploidy initiate neoplasia but are self-limiting as they become sources of cell lineage degradation. A differing process, cell–cell fusion, is required to energize and sustain aggressive and clinically relevant cancer. Through cell–cell fusion, likened to sexual reproduction, it is believed that parasitical cancer gains ecological fitness. Certainly, cell–cell fusion plays a substantial role in fertilization, tissue regeneration, and other physiological processes. But it is also a feature of pathological processes, including infectious disease and cancer. In cancer, cell fusion has been linked to disease progression, metastatic proliferation, and resistance to apoptosis and therapeutic drugs [101]. The mediating process appears to be a chronic inflammatory tissue reaction and it is along this interface that cell–cell fusion between cancer stem cells and bone marrow-derived cells and macrophages appear to facilitate cancer progression [102]. In a cognition-based frame, the entire issue is clarified. As tumor cells actively engage in niche construction, normal cells of all types become potential problem-solving tools for the neoplastic constituency. In these terms, tumorigenic latency becomes a function of a neoplastic recursive feedback loop within each specific cellular ecology. Successful proliferation relates to the problem solving circumstances of the specific self-referential neoplastic player and the degree to which collective normative cellular resources can be either recruited or organized in opposition.

When evolution or neoplasia are reevaluated as end results of self-referential agencies, both processes no longer constitute a random set. Perforce, cancer proliferation must be considered the action of a problem-solving constituency in participation and competition with other ecological players. In this manner, its actions become much like any parasitical infectious agency and then, in that respect, its urge to survive need not differ from any foreign agent in any localized tissue ecology. Any consideration of cancer through a model of infectious disease dynamics must extend beyond the limitations of the known associations between infectious agents and cancer [103]. For example, the possibility that tumorigenic stem cell origination is secondary to cell–cell fusion and horizontal gene transfer has been raised [104]. Biological interactions of that kind can be properly considered as a type of infectious interchange, no matter the exact etiology of cancer. Within that line of reasoning, if cancer is regarded as a type of self-aware parasitical entity, neoplastic cells should then be viewed as engaging in maladaptive behavior in the same manner as infectious entities that can begin to control any localized tissue ecology. In this context, the neoplastic maladaptive player might well be served beyond the standard reach of normal cells, according to its homeostatic needs, akin to any infectious agent. As normal cells respond by higher cellular activity and metabolic action to engage in countervailing niche construction as their ecological defense against neoplasia, as they might to any foreign intruder, they are inadvertently recruited to assist in creating and sustaining the preferential homeostatic milieu of the reproducing cancer cells. This is cancer’s privilege based on its ambiguous immunological status. Such action has been documented by tumor-associated macrophages in solid tumors [105]. In this sense, even if cancer is not considered a true parasite, its actions are an engagement within tissue ecologies as a form of parasitical homeostasis. It is recognized that the ‘host’ tissue environment is an active participant in cancer progression and metastasis [106]. It is also known that tumor invasion occurs within a tissue ecology when both general and neoplastic cells exchange bioactive products such as enzymes and cytokines. This permits the neoplasm access to resources to modify the local extracellular matrix, stimulate migration, and promote proliferation and survival in a manner that appears to be tissue specific [107, 108]. This may be the underlying narrative that permits the epithelial to mesenchymal transition that has been considered a prerequisite for tumor invasion and metastasis [109]. Under the terms of parasitical homeostasis, and at some threshold, the cascade of cytokines, inflammatory responses, and interleukins begins. This is subsequently co-opted by neoplasia. When these aspects are considered, it can be projected that it is not necessarily the presence of an abnormal cell lineage, per se, but those actions that sustain any resulting lineage. This new cellular ‘self’ can engage in natural cellular engineering, towards niche construction and resultant phenotype [110]. Therefore, the central issue for cancer becomes the context of phenotypic expression that is both competitive and collaborative within any tissue ecology vis-a-vis other constituencies at a site of neoplastic origin, rather than the intrinsic neoplastic cell.

Yet, no matter any explicit cause of CIN, there is an associated paradox. The widespread prevalence of CIN in cancer stands in contrast to evidence showing that aneuploidy induces a proteotoxic stress response and reduces cellular fitness [111]. However, this apparent discrepancy can be reasonably resolved by properly linking cancer to its correct evolutionary narrative. It is not dependent on fitness but is instead centered on cellular problem-solving imperatives that consistently derive from unicellular roots [8, 44]. Proliferation proceeds according to the homeostatic needs of the neoplastic cells, which is not directly related to fitness as judged by raw reproductive success. Therefore, instead of a Darwinian tautology in which survival defines fitness and, in turn, fitness is described by differential survival, cancer is a purposed self-referential entity whose direct aim is the maintenance of preferred homeostatic states. Survival is one its attendant parameters.

One advantage of proposing that cancer should be examined within a framework of infectious disease dynamics can be directed towards the acknowledged discrepancy between the presence of aneuploidy in tissue ecologies and oncogenesis. Not every instance of aneuploidy is associated with neoplasia. There are circumstances in which it serves a physiological purpose in reaction to stress [36]. Therefore, there is room for considering a cancer stem cell or lineage as a typical pathobiont, as a constituent whose rules of participation can be mutualistic or competitive/destructive depending on context. In this regard, it becomes similar to other infectious agencies such as *C*. *difficile*, whose differential impact is dependent on its status within its ecological community which it sometimes serves and at other times disrupts [112].

In this construct, any trigger for aneuploidy and genomic instability that might yield a mis-segregation error or some other tumorigenic response can result in a form of ecological dysbiosis. In that circumstance, since aneuploidy is common, cancer might be considered an aberrant attempt at repair from that epigenetic stress. In this frame, microenvironmental stress is the precipitating cause rather than an effect of oncogenesis. Under such conditions, the effect of an activating microenvironmental stress becomes a crucial trigger. For example, the result of that stress could be the expression of latent genomic inclusions (retroelements, ERVs) that can reside in the central genome. It can be considered that such an entity could act in a fashion similar to other cryptic pathobionts [113]. Under typical circumstances, these cryptic genetic elements might exist in apparent harmony within a tissue ecology. Even further, any might be in temporary service to its localized tissue ecology until a critical stressing event supervenes. Given the known association of cancer with some viruses, a critical trigger might be a viral incursion that might be context dependent. It has recently been found that such viral incursions can have a wide range of unanticipated effects. Not all of these are disadvantageous. Chénard et al. [114] report the viral donation of an immunity system into a freshwater filamentous cyanobacterium (*Nostoc* sp.) to encode a functional CRISPR array and a proteobacterial DNA polymerase. Viral incursion might therefore facilitate CIN by contributing to the ambiguous immunological status of infected cells, permitting CIN to be tolerated rather than expunged. At an initial stage, a virus or viral component might act as a typical communal pathobiont, but still triggering aneuploidy and genomic instability that is not initially connected to recognizable pathological outcomes. A subsequent event might be the further required trigger that leads to neoplastic dissemination. This would account for the variable latency typical of some cancers. For example, an epiphenomenon might trigger previously incorporated latent intra-genomic retroelements that had been down regulated and had remained quiescent. Such complex inter-reactions between genomes, endogenous retrovirus activation, and the microbiome within tissue ecologies are in fact known [115].

## Alternatives for treatment

**Fig. 1:**
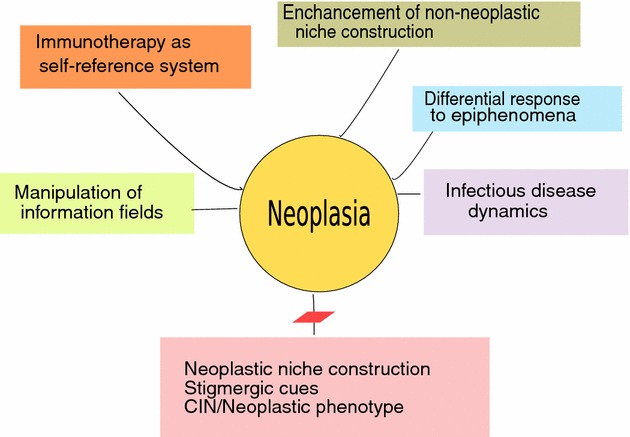
Potential targets of cancer therapy in CBE

Although it would be readily agreed that viewing neoplasia as a self-referential agency might not initially change the molecules or drugs used to treat cancer or their targets, over time, it would surely change the frame of reference in which they are employed. Greaves and Maley [39] have stressed the importance of reiterative processes of clonal expansion supported by genetic diversification. In the past, those dynamics are considered to be inherently Darwinian in character in which clonal destruction inadvertently enables selection pressure for the expansion of treatment- resistant clonal variations. In CBE, the evolutionary narrative extends beyond a search for methods to kill cells or suppress proliferation towards explicitly confronting cancer as a problem-solving agency that relies upon its own information space for its advantage (Fig. 1).

Any achieved success may have its best opportunities based upon the unique characteristics of clonal lineages that privilege cancer versus other cells on the one hand, or disadvantage it on another. For example, the lack of cancer lineage heterozygosity and its clonal origins, despite cell–cell fusions, might be expected to have its own types of impaired resistance to other threats. This disadvantageous aspect of clones is known through our clonal cultivation of the seedless fruit varietals that human consumers favor. Bananas are such a clonal cultivar and have been subject to devastating fungal epidemics that have nearly wiped out entire lineages, such as the Gros Michel variety [116]. Therefore, it is reasonable to assume that cancer may be able to co-opt resistance mechanisms through the backtracking of evolution towards the prokaryotic toolkit but it still likely that it has countervailing aspects of exclusive susceptibility that might be simultaneously exploited.

Within an alternate framework of CBE, the approach to cancer treatment could be undertaken in a variety of ways:

1. *Extirpation of cancer along immunological lines.* T cell enhancement can be examined through the lens of both neoplasia and T cells as a self-referential problem-solving agencies and not just reactive and unknowing immunological participants.
2. *Manipulation of the tumor microenvironment considers cancer as a problem*-*solving agency of niche construction using stigmergic cues*. Countering such a skillful player would involve the disruption of phenotypic exploration of the microenvironment, such as deprivation of resources, or blocking the recruitment of other cellular constituencies.
3. *Encourage counter*-*reactive niche construction by the non*-*neoplastic constituents of the localized tissue ecology*. This is a method of redirecting the tumor microenvironment towards a ‘correct’ homeostatic status by which all the constituents of an entire tissue ecology are pressed towards a normative information set. This might be best accomplished by winnowing those cells that are attuned to a differing information background. This is cellular engineering directed toward an adaptive tissue-wide phenotype and has been the subject of experimentation [117].
4. *Disruption of CIN permits fewer phenotypes and obstructs the proliferation of clonal lineages.*
5. *Alteration of the information field that cancer uses to guide tumor proliferation and induce non*-*cancer cell collaboration or trading of resources.* Many methods such as ultrasound or heat have been tried but have been directed towards tumor destruction [118, 119]. These might be differently directed towards a new frame in which distortion of cancer’s connection with its information space is the primary goal. Either the information space or its tools might be interrupted. For example, recent research has demonstrated that cancer utilizes exosome mediated transport in a unique manner to assist in tumor proliferation, viral exchange, microRNA transport, or exchange of signaling molecules or their breakdown products for communication of information among cancer cells and to aid the cancer community [120].
6. *Alteration or disruption of stigmergic cues that neoplasia uses to assist in collaboration in tumor microenvironments through manipulation of morphogenic fields.* Such fields are large-scale systems of physical properties that may store patterning information and guide tissue repair and cancer suppression. The use of endogenous bioelectric signals to exert control has been proposed [121].
7. *Seek differential responses to a broad range of epiphenomena.* As a differing self-referential agency, cancer will therefore differ in some crucial aspects from normal cells in its response to epigenetic stresses. Such idiosyncrasies might be exploited [122].
8. *Explore a parasite of parasite model.* Prior reports have noted the anti-tumor effects of some infections. For example, malaria has been shown to suppress tumor formation via induction of innate and adaptive anti-tumor responses in a mouse model [123]. Recent research has uncovered a surprisingly wide range of circumstances regarding parasitical infection, even among other parasites. For example, the parasitical series of infectious interactions between amoeba and an infecting giant Lentille virus is instructive. This giant virus is in turn, targeted by an infectious virophage (Sputnik 2), which is then further infected by transpovirons (bits of parasitic DNA) [124].
9. *Further investigate the infectious transmission of some cancers.* Although still believed to be rare and initially thought to be confined to dogs and Tasmania devils, a recent report of transmissible cancer in several mollusk species has been documented [125]. Furthermore, one of the mollusk species transmitting that cancer is not itself susceptible to that cancer. A potential implication is that a pathogenic cancer cell is itself an infectious form originating in one individual and spreading as a single-celled organism acting as a self-referential entity with its own particular purposes [75]. It is of further interest that this interaction has been shown to be characterized by a lack of diversity in Major Histocompatibility Complex genes [126]. These are known to play a significant role in immune surveillance, infectious disease susceptibility, and self/non-self recognition that have been associated with the spread of contagious cancers. There has been recent further evidence that the infectious spread of cancer extends beyond the theoretical. In 2015, a case of transmission of cancer from an infectious tapeworm, *Hymenolepis nana,* to a human was proved [127].

Successfully overcoming the protean manifestations of cancer requires a grounded understanding of its discrete cellular nature and the exact circumstances of its cellular opposition. If it is acknowledged that the success of a neoplastic agency extends beyond a selection imperative, a new frame might be the inspiration for productive reexamination of some prior successful therapies. Instead of selection and simple rates of proliferation as targets, self-referential identity, cellular engineering as active niche construction, and cellular problem-solving capacities are its touchstones. In each instance, the common theme towards extirpation or control becomes the disruption of the cognitive toolkit of cancer. 

## Perspective

 It is certainly not typical to ask whether or not cancer has a purpose. However, in the circumstance in which neoplasia is recognized as a self-referential agency, that question can be proposed. In infectious disease dynamics, scientists are accustomed to judging the life cycles and varying hosts of many microorganisms that seek their transfer by being shed, excreted or consumed by predators. When all multicellular eukaryotes are considered holobionts, any self-referential agency might view the death of the current organism in which it is a participant as just a stage towards its differing disposition. Animals and plants shed cells or are consumed by the next organism. Such directions might serve the cell as well or better than its current status. This may be the more so if immortal cancer cell lineages are triggered by viral inclusions that may find their preferential self-referential status in the successor microenvironment. Cancer’s unique ability is its access to a normally excluded evolutionary toolkit that enables the advantaged insinuation and domination by cancer cells throughout an extensive range of mixed microbial/cellular ecologies. It is proposed that in cellular terms, this empowerment and restraint is the cusp of cellular creativity. Therefore, it can be considered that neoplasia has its own form of creativity when placed within a permissive environment. Within its milieu, cancer is a creative agency in which a differential karyotype, ultimately expressed as phenotype, empowers a path towards both fitness and self-defined homeostatic equipose. This proceeds beyond merely random occurrences through a determined exploration of information space. Cancer can then be seen as a narrative based on its own self-directed purposes that extends beyond just genetic diversity and cellular proliferation as only related to Darwinian selection (Table 2).

**Table 2:** Summary of the differential characteristic features of neoplasia within a self-referential evolutionary frame compared to a NeoDarwinian model

Cognition-based evolution	NeoDarwinian selection model
Self-referential cellular cognition	Differential fitness driving clonal selection
Information and communication dependent	
Problem-solving to maintain homeostasis	
Non-random communication/problem solving	Stochastic mutations
Natural cellular engineering	Differential fitness/survival
Stigmergy/niche construction as problem-solving	Niche construction via clonal selection,
Reverse evolution/hyperadapability	Random mutation/selection
Privileged cellular participant/observer status	Passive selection driven participation
Phenotype as self-referential environmental exploration	Phenotype as a result of clonal selection
Natural selection is a post facto filter	Natural selection shapes neoplastic phenotype
Genes/CIN as informational tools and a flexible response system	Random genetic mutations drive neoplasia Genes as code
Integrated proteomics/transcriptomics	Oncogenes
Primacy of immunology to sustain self-reference	Immunology as a secondary phenomenon
Deterministic cellular creativity solves problems	Fitness/selection; stochastic variables
Initiates as self-directed pathobiont	Random replication error
Parasitical homeostasis	

When understood to emanate from a state in which basal cellular cognition is its proper frame, each separate clonal outgrowth from any initial neoplastic lineage is assumed to have its own self-referential state. It occupies its own information space and assigns its particular decision matrix of Bohmian implicates and explicates according to its own ‘self’. Therefore, it participates in each ecology as its own independent and self-referential niche participant. Critically, the pervasive information space of any cancer stem cell or proliferating neoplasia is different from other cells in any tissue ecology. The karyotypic heterogeneity of cancer cells facilitates its escape from any standard cellular adjudication of information space. Yet still, those neoplastic cells have emanated from within their initiating environment. Thus, cancer karyotype energizes its unique status by permitting connection to the common information space of any localized microenvironment, and furthering itself through its own unique self-referential status that releases it from typical cellular constraints. Further yet, its first successes are propelled by limited immunological surveillance. Therefore, it is no longer surprising that its first manifestation is as a useful ecological player and may remain so for some propagative iterations thereafter. Neoplasia thereby engages in its own specific and extensive cellular entanglement within any mixed tissue ecology that it occupies. Within that ecology, genes are utilized as flexible tools. Neoplasia can therefore explore local and distant environments by utilizing CIN as pathways toward alternate phenotypes. Other cells become untoward recruits during repeated rounds of niche construction that extends beyond raw selection, and instead, remains centered within the maintenance and furtherance of its own preferential homeostatic status in apposition to environmental and epigenetic stresses. Just like all cells, neoplastic cells remain in connection with their unicellular roots. However, as opposed to a typical differentiated cell, the more flexible cancer cell, in the absence of normal checkpoints, utilizes an invigorated form of reverse evolution for coping with epigenetic stresses. This enables the neoplastic clonal lineage to reach backward into its cellular toolkit to maintain preferential homeostasis. It directs this flexible backwardization towards its phenotypic map through processes of natural cellular engineering, similar to all cells. This proceeds via stigmergic cues that enables purposeful niche construction. Pluripotential capacity achieved through rapid CIN and phenotypic variation energizes its expansion in favored niches within localized or distant cellular ecologies. As a cellular constituent within such ecologies, it communicates and cooperates just as all cells do. However as a privileged entity, it out engineers other participants. Above all else, within its cellular domain, neoplasia is sanctioned as an exceptional problem-solving agency granted privilege through its exclusive self-referential participant/observer status.

In such cellular dynamics, it can be assumed that each participant in any mixed microbial/innate cellular ecology is in some way being served within its niche, even if its position relative to others for scarce resources is disadvantaged. Parasitism can only succeed if something is left for the other constituents of the affected ecology. Absent that impulse, it destroys itself. Therefore, a pertinent question must be posed. What might cancer, as a self-referential agency, be seeking beyond reproduction of a clonal lineage? As with all cells, that answer emanates from its self-referential capacity that is directed to the maintenance of homeostatic equipoise. As a practical implication, it becomes important to consider which cellular constituency is being served by the proliferation of cancer? What is the specialized configuration of information space utilized by neoplastic cells that they can discern by virtue of their unique observer/participant/status as an independent ‘self’? From these differing vantage points, novel cancer treatments might devolve.

When considered in this manner, neoplasia can be viewed as the differing expression of normal ecological processes co-opted by a variant ‘self’ that can now act as a parasitical constituency. Cancer expression and its limits are therefore a function of a parasitical cell lineage vis-a-vis normal cells. That variant ‘self’ is purposed towards the maintenance of its own parasitical homeostasis in which the range of reinforcing behaviors is extraordinarily broad. Whether or not this parasitical constituency is overtly harmful or ‘well-adapted’ towards its host is highly context dependent [128]. Therefore cancer is best regarded as acting as a quasi-autonomous organoid, as a form of ‘parasitical pathobiont’, in which its success might be illuminated by comparisons to infectious disease dynamics.

There is a particular facet of a cognitive framework for cancer that may offer an answer to an evolutionary puzzle. Evolution is a narrative of both function and form. Both must be considered in any evolutionary cancer schema. Certainly, co-option of physiology and metabolism through recruitment of constituencies for cancer niche construction or the evolutionary backtracking of cancer cells are both explicit paths that link functional aspects of evolution to cancer dynamics. The issue of form in cancer can be addressed by regarding cancer as atypical phenotypic expression of an abnormal karyotype that proliferates within a localized tissue ecology. This phenotype is a cancer’s cellular solution to its own self-referential stresses by which it attempts to settle environmental ambiguities within its own proscriptions. Within evolutionary terms, there is a major issue is any attempt to devise a robust pathway from genes to actual forms [129]. That path remains unclear but cancer lineages may be instructive. Cancer phenotypes engage in cellular engineering and niche construction through self -referential means that are largely deterministic within any tissue ecology. This is an example of cellular impulses becoming oncogenic form whose results extend beyond stochastic genetic mutation. It is, instead, an example of cellular problem-solving activity. Although neoplastic clonal lineages are often compared to alternative phenotype, oncogenic structures are not typically considered an explicit example of phenotypic form in the macro sense. Nonetheless, they should definitely be considered in that manner even if that form makes no discrete sense within our own human frame. Cancer has its particular architecture and succeeds with it just as macroscopic phenotype does. Therefore, it can be considered that cancer is an expression of general evolutionary mechanisms by which phenotype bubbles up from cellular proclivities to become eventual perceptible form in continuous reaction to epigenetic stress. It accomplishes this through means that extend beyond random variation and selective filtering. In the alternate frame of CBE, cancer becomes evolutionary self-organization gone askew. In the normal state, phenotype arises by the concatenated process of the continual consensual recruitment of constituencies in localized tissue ecologies through natural cellular engineering. Cancer is similarly purposed towards self-directed niche construction that ultimately yields phenotype. Like other cells, the neoplastic stem cell achieves its form with genes and other cellular components as its flexible tools to meet environmental stresses in which there is no requirement for any absolute one-to-one relationship.

## Conclusion

It is maintained that there are six identifiable biological capacities that are acquired during the development of human neoplasms [130]. These include sustaining proliferative signaling, resistance to growth suppressors, eluding apoptosis enabling cancer lineage immortality, the induction of angiogenesis, and tumor invasion/metastasis. Each of these is bolstered by genomic instability that promotes cancer niche construction. By co-opting resources, by reprogramming energy metabolism to support their own proliferation, through the evasion of immunological destruction, by recruiting other cellular constituents, and through the flexible backwardization of its evolutionary toolkit, cancer lineages enact preferential tumor microenvironments. Each of these is used to advantage through the cellular mechanisms of niche construction and stigmergy common to all cellular networks.

In evolution, there are two context-dependent cellular/microbial functions that define all eukaryotic tissue ecologies that are often overlooked. The first is, ‘Who is serving and who is being served?’ The other, ‘Who is observer and who is participant?’ [8]. In CBE, the answer is always enacted at the cellular level at which constituencies act at every scope and scale that together make the whole. This is also always assessed through the self- referential status of each of the particular constituents, in any tissue ecology, and therefore, within any tumor microenvironment. At all times, each and every self-referential participant is both subject and object in circumstances in which all must use ambiguous information to settle their own range of implicates towards self-directed explicate solutions. The next step in cancer research is to better understand this differing stance.

## Abbreviations

CBE: cognition based evolution
LECA: last eukaryotic common ancestor
